# Randomized, double-blind, crossover study comparing DFN-11 injection (3 mg subcutaneous sumatriptan) with 6 mg subcutaneous sumatriptan for the treatment of rapidly-escalating attacks of episodic migraine

**DOI:** 10.1186/s10194-016-0717-7

**Published:** 2017-02-07

**Authors:** Roger K. Cady, Sagar Munjal, Ryan J. Cady, Heather R. Manley, Elimor Brand-Schieber

**Affiliations:** 1 Clinvest/A Division of Banyan Inc., 3805 S Kansas Expy, Springfield, MO 65807 USA; 2 Dr. Reddy’s Laboratories Ltd., 107 College Road East, Princeton, NJ 08540 USA

**Keywords:** Episodic, Migraine, Rapidly-escalating, Treatment, Sumatriptan, Subcutaneous

## Abstract

**Background:**

A 6-mg dose of SC sumatriptan is the most efficacious and fast-acting acute treatment for migraine, but a 3-mg dose of SC sumatriptan may improve tolerability while maintaining efficacy.

**Methods:**

This randomized, double-blind, crossover study compared the efficacy and tolerability of 3 mg subcutaneous (SC) sumatriptan (DFN-11) with 6 mg SC sumatriptan in 20 adults with rapidly-escalating migraine attacks. Eligible subjects were randomized (1:1) to treat 1 attack with DFN-11 and matching placebo autoinjector consecutively or 2 DFN-11 autoinjectors consecutively and a second attack similarly but with the alternative dose (3 mg or 6 mg).

**Results:**

The proportions of subjects who were pain-free at 60 min postdose, the primary endpoint, were similar following treatment with 3 mg SC sumatriptan and 6 mg SC sumatriptan (50% vs 52.6%, *P*  =  .87). The proportions of subjects experiencing pain relief (*P*  ≥  .48); reductions in migraine pain intensity (*P*  ≥  .78); and relief from nausea, photophobia, or phonophobia (*P*  ≥  .88) with 3 mg SC sumatriptan and 6 mg SC sumatriptan were similar, as were the mean scores for satisfaction with treatment (*M*  =  2.6 vs *M*  =  2.4, *P*  =  .81) and the mean number of rescue medications used (*M*  =  .11 vs *M*  =  .26, *P*  =  .32). The most common adverse events with the 3- and 6-mg doses were triptan sensations — paresthesia, neck pain, flushing, and involuntary muscle contractions of the neck — and the incidence of adverse events with both doses was similar (32 events total: 3 mg, *n*  =  14 [44%]; 6 mg, *n*  =  18 [56%], *P*  =  .60). Triptan sensations affected 4 subjects with the 6-mg dose only, 1 subject with the 3-mg dose only, and 7 subjects with both sumatriptan doses. Chest pain affected 2 subjects (10%) treated with the 6-mg dose and no subjects (0%) treated with the 3-mg dose of DFN-11. There were no serious adverse events.

**Conclusions:**

The 3-mg SC dose of sumatriptan in DFN-11 provided relief of migraine pain and associated symptoms comparable to a 6-mg SC dose of sumatriptan. Tolerability was similar with both study medications; DFN-11 treatment was associated with fewer triptan sensations than the 6-mg dose. DFN-11, with its 3-mg dose of sumatriptan, may be a clinically useful alternative to higher-dose autoinjectors.

## Background

Migraine is a chronic neurologic disease characterized by recurrent episodes of headache associated with a wide array of disruptive symptoms, including photophobia, phonophobia, nausea, and/or vomiting [1, 2]. It is the most common neurological disease for which people seek medical consultation, affecting about 12% of adults in the United States, three quarters of them women [3–5]. Nearly all migraineurs (93%) have some degree of attack-related impairment, and more than half (54%) are severely impaired by their attacks [3]; among women, migraine ranks in the top 10 causes of disability worldwide [6]. Because migraine is heterogeneous and often spans decades of patients’ lives, clinical presentations and acute treatment needs can vary considerably over time, and patients often require different formulations of acute medications to optimize treatment outcomes [7]. This is particularly true when attacks are severe, accompanied by nausea and vomiting, or rapid in onset, and parenteral formulations are needed to control symptoms.

Sumatriptan — the first, most widely studied, and most frequently prescribed member of the “triptan” class of medications [2, 8, 9] — is available as a subcutaneous (SC) injection, intranasal spray, oral tablet, and, in some countries, a rectal suppository. The 6 mg SC injection has long been considered optimal for acute therapy of migraine [2]; in large double-blind, randomized, placebo-controlled clinical trials, 70% of subjects with moderate to severe attacks experienced pain relief within 1 h of dosing, and 50% were pain-free [10, 11]. The rapid onset of action of SC sumatriptan is especially important for patients whose attacks become disabling soon after onset [12].

Despite its well-established efficacy and rapid onset of action, sumatriptan 6 mg SC injection has a suboptimal tolerability profile. Many patients (42%) experience triptan sensations [13], such as paresthesia and chest symptoms, that appear to be dose-related [14]. Concerns about drug-related adverse events (AEs), which have caused two thirds of migraine patients to delay or avoid taking a prescription medication, may help to explain why fewer than 10% of eligible migraine patients use the SC formulation of sumatriptan to treat their disease [15]. These concerns can be particularly important in patients with various types of medical conditions (eg, cardiovascular and cerebrovascular disease and some psychiatric illnesses) and/or treated with various medications (eg, monoamine oxidase inhibitors and selective serotonin reuptake inhibitors) [9]. Based on previous research [16], we hypothesized that a formulation using a lower doses of sumatriptan may improve safety and tolerability while maintaining efficacy similar to 6 mg SC sumatriptan, and that a 3-mg dose may be sufficient in many patients. The objective of this exploratory study was to compare the efficacy and tolerability of 3 mg SC sumatriptan (using DFN-11 and matching placebo autoinjectors consecutively) with 6 mg SC sumatriptan (using 2 DFN-11 autoinjectors consecutively) for the acute treatment of episodic migraine attacks.

## Methods

This was a double-blind, crossover, pilot study conducted at a single study center (Clinvest; Springfield, MO). The study compared a 3-mg SC dose of sumatriptan with the commonly prescribed 6-mg SC dose in 19 subjects. The protocol was approved by the Sterling Institutional Review Board, and the study was conducted in compliance with good clinical practice and in accordance with the ethical principles set forth in the Declaration of Helsinki. Prior to the initiation of any study-specific procedures, investigators explained the nature of the study to the subjects, and subjects provided informed consent. The first subject was enrolled on 15 September 2015, and the study was completed on 14 April 2016. Additional information about this trial is available online at ClinicalTrials.gov (Identifier: NCT02571049).

### Study conduct

To participate, subjects recruited from the clinic and general population had to satisfy a range of inclusion and exclusion criteria (Table 1). The research coordinator and investigator initially evaluated eligibility and obtained informed consent. Final eligibility was determined by the investigator, and the research coordinator completed the enrollment process.

**Table 1:** Inclusion and exclusion criteria

Inclusion	Exclusion
ICHD-3-beta episodic migraine^a^	Unable to distinguish migraine from other primary headache conditions
Onset of migraine before age 50	15 or more headache days per month
≥ 3-month history of 2–8 attacks/month; ≥ 75% of attacks progress to moderate or severe pain and/or nausea within 2 h (ie, rapidly-escalating)	Opioid usage > 10 days in the 30 days before screening
Acute headache medication on ≤ 14 days/month in the 3 months prior to enrollment	Use of MAO-A inhibitors within 28 days of randomization
No sumatriptan injections for ≥ 3 months	History of hemiplegic/basilar migraine; epileptogenic conditions; symptoms or signs of ischemic cardiac, cerebrovascular or peripheral vascular syndromes, or uncontrolled hypertension
If taking migraine prophylaxis, stable for 30 days before and throughout the study	Drug or alcohol abuse within the previous 2 years
Females: negative urine pregnancy test at screening, effective birth control, or surgically sterile or postmenopausal for ≥ 1 year before enrollment	Systemic disease or neurological or psychiatric condition^b^
Willing to read and comprehend written instructions and internet access for electronic diary	Investigational medication ≤ 30 days before randomization
Sign informed consent document	Positive urine drug screen for recreational drugs, marijuana, or prescription drugs not explained by stated concomitant medications
	Clinical laboratory or ECG abnormality
	Fridericia’s corrected QT interval > 450 msec
	Creatinine > 2 mg/dl; serum total bilirubin > 2.0 mg/dL
	Serum AST, ALT, or alkaline phosphatase > 2.5 times ULN
	Rebound headache from caffeine usage^b^

*ICHD* International Classification of Headache Disorders, *MAO-A* monoamine oxidase A, *ECG* electrocardiogram, *AST* aspartate aminotransferase, *ALT*, *ULN* upper limit of normal

^a^With or without aura

^b^Contraindicated participation in the opinion of the investigator

The study consisted of 4 visits: screening, randomization, treatment crossover, and end-of-study. Subjects treated up to 2 attacks within 4 weeks. At the screening visit, subjects provided a detailed medical and headache/migraine history, a physical examination was performed, laboratory assessment samples (ie, hematology, serum chemistry, urine analysis, serology, urine drug screen) were obtained, inclusion/exclusion criteria were verified, and a 12-lead electrocardiogram (ECG) was completed. Eligible subjects returned to the site with 14 days of the screening visit and were trained in the appropriate use of the autoinjector device and given printed instructions to take home that reinforced correct DFN-11 administration.

Subjects were divided into 1 block and randomized (1:1) to receive 3 mg SC sumatriptan (using DFN-11 and matching placebo autoinjectors consecutively) or 6 mg SC sumatriptan (using 2 DFN-11 autoinjectors consecutively) in a crossover design and were dispensed the first treatment in the sequence. The randomization scheme was generated by study personnel with no subject interaction or other monitoring roles using an online tool (http://www.randomization.com); it was provided to the drug packing company, which used it to prepare and pre-label the kits with sequential numbers. The research coordinator was instructed to dispense the lowest kit number available.

Diary instruction was provided, and subjects were encouraged to treat an attack within 1 h of onset of headache. They were asked not to use rescue medication until at least 120 min postdose. After subjects treated the first attack, they returned to the clinic for re-evaluation and treatment crossover. Before treating a second attack, subjects had to be pain-free for a minimum of 24 h. The end-of-study visit occurred within 7 days after treatment of the second attack or within 4 weeks of randomization, whichever came first. At the end-of-study visit, the subject’s diary information was reviewed, and all end-of-study visit procedures were performed.

Adverse events were monitored from the time subjects gave informed consent until the follow-up visit; physical examinations, vital sign measurements, ECGs, and laboratory assessments were performed at designated visits throughout the study period.

### Assessments

#### Efficacy

The prospectively defined primary efficacy endpoint was the proportion of subjects reporting freedom from pain at 60 min postdose; pain freedom was defined as a headache pain severity rating of 0 (no pain). Secondary endpoints included pain relief, pain freedom, and headache severity at 30, 60, 90, and 120 min postdose, proportion of subjects with relief of associated migraine symptoms (ie, nausea, photophobia, and phonophobia) at 60 min postdose, subject satisfaction with treatment, and use of rescue medication from 2 to 24 h postdose. Pain relief was defined as a 1-point reduction in headache pain intensity, which was rated on a 4-point Likert scale where 0  =  no pain, 1  =  mild pain, 2  =  moderate pain, and 3  =  severe pain. Relief of the associated symptoms of nausea, photophobia, and phonophobia was defined as a 1-point reduction in symptom severity where 0  =  no symptom, 1  =  mild, 2  =  moderate, and 3  =  severe. Subject satisfaction with treatment was based on a 7-point Likert Scale, with 1 being very satisfied and 7 being very dissatisfied. Use of rescue medication was calculated by totaling the number of rescue medications used from 2 to 24 h postdose.

The primary and most of the secondary endpoint data were derived from an online headache e-diary. Daily diary assessments included onset and duration of headache, severity of pain at the time of onset and at treatment, acute headache pain medication(s) usage, study drug usage, associated symptoms, unusual symptoms, and subject satisfaction with treatment.

#### Tolerability

Tolerability was assessed by comparing AEs occurring up to 24 h postdose. Safety measures included AEs, physical examinations, vital signs, ECG readings, and laboratory assessments (hematology, serum chemistry, urine analysis). Adverse events were coded with the Medical Dictionary for Regulatory Activities (MedDRA, version 17.0) and classified by date of onset, duration, frequency, severity, and relationship to study medication. The classification of AEs as triptan sensations was determined by principal investigator. Spontaneously reported AEs were recorded for up to 5 days after the last dose of study drug; AEs determined as serious were recorded for up to 30 days.

### Statistical analysis

All statistical tests were 2-tailed, and an alpha of .05 was used to determine statistical significance. Descriptive statistics established baseline characteristics and AE frequency. Data for the primary endpoint was analyzed via chi-squared analyses; the secondary outcome measures were analyzed with a 2-tailed Repeated Measures analysis of variance (ANOVA), chi-squared, and/or independent or dependent t-tests, as appropriate. All ANOVAs were followed by univariate post hoc tests, as appropriate, and multiple comparison adjustments were made as needed. A last observation carried forward method was used to impute missing values within a single headache diary attack.

Efficacy analyses were performed on a modified intent-to-treat population, which included all randomized subjects who received at least 1 dose of study drug and obtained at least 1 endpoint measurement (mean change from baseline in the number of hours with headaches) after treatment. The safety population included all randomized subjects.

## Results

**Fig. 1:**
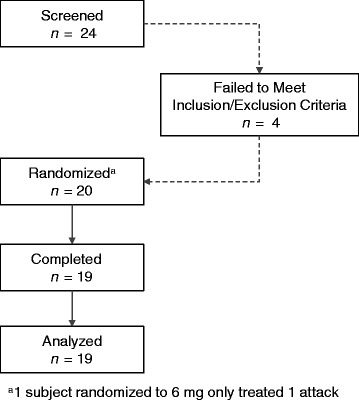
Subject disposition

Twenty-four subjects were screened, 20 subjects were randomized (1 never treated), and 19 subjects treated at least 1 migraine attack (Fig. 1). The study population was 80% female and 95% Caucasian, with a mean age of 39.8 years. At baseline, subjects were experiencing 5 migraine days and 6.8 headache days per month on average (Table 2).

**Table 2:** Baseline demographics

Variable	Value
Gender *n* (%)	
Male	4 (20)
Female	16 (80)
Age (years)^a^	39.8 (10.4)
Range	19–61
Race *n* (%)	
Caucasian	19 (95)
Hispanic	1 (5)
Headache Characteristics^a^	
Migraine Days/Month	5.0 (3.7)
Headache Days/Month	6.8 (1.7)

^a^Values are mean (SD)

**Fig. 2:**
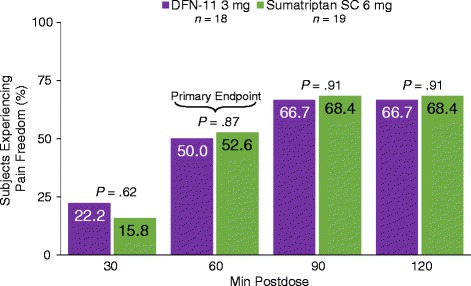
Pain freedom at 30, 60, 90, and 120 min postdose^a,b^. Legend: SC, subcutaneous. ^a^Mean (SD) predose pain intensity average was 2.00 (.58). ^b^1 subject randomized to 6 mg only treated 1 attack

The proportion of subjects who were pain-free at 60 min postdose, the primary efficacy endpoint, was 50.0% for 3 mg SC sumatriptan and 52.6% for 6 mg SC sumatriptan (*P*  =  .87), as shown in Fig. 2. Pain-free responses to 3 mg SC sumatriptan and 6 mg SC were already apparent at 30 min (22.2% vs 15.8%, respectively; *P*  =  .62). At 90 min, 66.7% vs 68.4% of subjects were pain-free (*P*  =  .91), and these responses were sustained to 120 min postdose (66.7% vs 68.4%; *P*  =  .91).

**Fig. 3:**
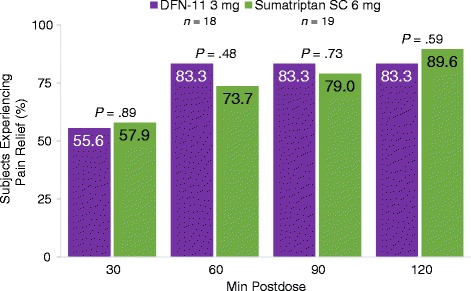
Pain relief at 30, 60, 90, and 120 min postdose^a^. Legend: SC, subcutaneous. ^a^1 subject randomized to 6 mg only treated 1 attack

**Fig. 4:**
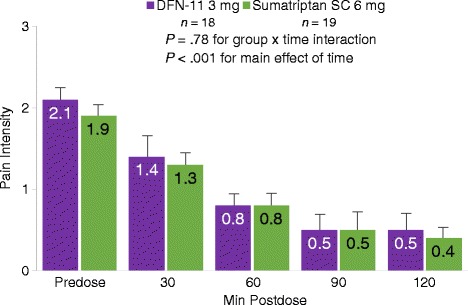
Pain intensity at predose, 30, 60, 90, and 120 min postdose^a,b,c^. Legend: SC, subcutaneous. ^a^1 subject randomized to 6 mg only treated 1 attack. ^b^Repeated Measures ANOVA revealed an insignificant group and time interaction: F(2.43, 85.02)  =  .61, *P*  =  .78. ^c^A significant main effect of time was found for pain intensity scores: F(2.43, 85.02)  =  .61, *P*  <  .001

Eighty-three percent of subjects experienced pain relief with 3 mg SC sumatriptan at 60, 90, and 120 min postdose; the corresponding pain relief rates with the 6-mg dose, 73.7% (*P*  =  .48), 79.0% (*P*  =  .73), and 89.5% (*P*  =  .59), were not significantly different at any timepoint (Fig. 3). With a mean (SD) predose pain intensity of 2.1 (.6), reductions in migraine pain intensity after treatment with 3 and 6 mg SC sumatriptan were comparable at all time points (*P*  =  .78) over the 2-h assessment period (Fig. 4).

**Fig. 5:**
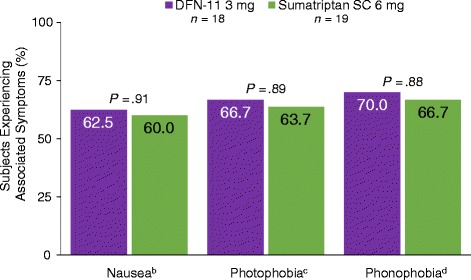
Proportions of subjects experiencing relief from symptoms associated with migraine at 60 min postdose.^a^ Legend: SC, subcutaneous. ^a^1 subject randomized to 6 mg only treated 1 attack. ^b^3 mg *n*  =  8; 6 mg *n*  =  10. ^c^3 mg *n*  =  9; 6 mg *n*  =  11. ^d^3 mg *n*  =  10; 6 mg *n*  =  9

At 60 min postdose (Fig. 5), there were no significant differences between 3 and 6 mg SC sumatriptan in the proportions of subjects who experienced relief from nausea (*P*  =  .91), photophobia (*P*  =  .89), or phonophobia (*P*  =  .88). The mean number of rescue medication doses used over the course of the study period was not significantly different when subjects treated with 3 mg or 6 mg SC sumatriptan (*M*  =  .11 vs *M*  =  .26). Subjects were similarly satisfied with either treatment, with no significant difference in mean scores for 3 and 6 mg SC sumatriptan (*M*  =  2.6 vs *M*  =  2.4).

### Tolerability

In the safety population (*N*  =  20), 80% of subjects (16/20) experienced a total of 50 AEs. The overall incidence of AEs with DFN-11 (.72) and 6 mg SC sumatriptan (.74) was comparable (*P*  =  .97), and the most common AEs were triptan sensations: paresthesia (30%, 6/20), neck pain (20%, 4/20), flushing (10%, 2/20), chest pain (10%, 2/20), involuntary muscle contractions of the neck (10%, 2/20), and vomiting (10%, 2/20). As shown in Table 3, there were no significant differences in the frequency (14 vs 18 events, *P*  =  .60), duration (27 vs 64 min, *P*  =  .43), and severity (1.29 vs 1.28 [0–3 scale], *P*  =  .97) of triptan sensations with DFN-11 and 6 mg SC sumatriptan. Of the 12 subjects who reported triptan sensation AEs, 7 subjects experienced them with DFN-11 and 6 mg SC sumatriptan, 1 subject experienced them only after DFN-11, and 4 subjects experienced them only following 6 mg SC sumatriptan. There were no meaningful changes from baseline in vital signs, ECGs, or laboratory assessments, and no serious AEs were reported.

**Table 3:** Triptan sensations after treatment with 3 and 6 mg SC sumatriptan

	3 mg SC sumatriptan			6 mg SC sumatriptan		
	Event frequency *n* (%)	Duration^a^ Min	Severity^a,b^ (1–3)	Event frequency *n* (%)	Duration^a^ Min	Severity^a,b^ (1–3)
Paresthesia	5 (15.63)	13.0 (.01)	1.20 (.45)	4 (12.50)	20.0 (.01)	1.0 (0.0)
Neck Pain	4 (12.50)	12.0 (.01)	1.25 (.50)	3 (9.38)	11.0 (.00)	1.0 (0.0)
Flushing	2 (6.25)	25.0 (.01)	1.50 (.71)	2 (6.25)	11.0 (.01)	1.50 (.71)
Muscle Contractions (Neck)^c^	1 (3.13)	44 (N/A)	1.0 (N/A)	2 (6.25)	47 (N/A)	1.50 (.71)
Chest Pain	0 (0)	—	—	2 (6.25)	374 (.35)	2.0 (1.41)
Disorientation	0 (0)	—	—	1 (3.13)	19 (N/A)	1.0 (N/A)
Dizziness	0 (0)	—	—	1 (3.13)	20 (N/A)	1.0 (N/A)
Myalgia	0 (0)	—	—	1 (3.13)	44 (N/A)	1.0 (N/A)
Tinnitus	0 (0)	—	—	1 (3.13)	41 (N/A)	1.0 (N/A)
Vomiting	0 (0)	—	—	1 (3.13)	41 (N/A)	1.0 (N/A)
Hyperhidrosis	1 (3.13)	38 (N/A)	1.0 (N/A)	0 (0)	—	—
Malaise	1 (3.13)	130 (N/A)	2.0 (N/A)	0 (0)	—	—
Total	14 (44)^d^	27 (33.12)^e^	1.29 (.47)^f^	18 (56)	64 (167.10)	1.28 (.57)

*SC* subcutaneous

^a^Mean (SD)

^b^Assessed on a 3-point Likert scale: mild  =  1, moderate  =  2, and severe  =  3

^c^Involuntary

^d^ *P*  =  .60 vs 6 mg

^e^ *P*  =  .43 vs 6 mg

^f^ *P*  =   .97 vs 6 mg

## Discussion

Migraineurs consistently rate rapid onset of pain relief and tolerability of treatment among the most important attributes of acute migraine medication [17, 18]. With an onset of action of approximately 10 min and unparalleled relief of migraine headache and associated symptoms [14], SC sumatriptan has been the most effective acute migraine therapy since its introduction nearly 25 years ago [2]. The primary limitations to its use as an acute therapy are medication-related AEs. Understanding the tolerability of treatment is important as to why migraine patients settle for less effective alternatives [16]. For many, the relatively high likelihood of experiencing triptan sensations is a barrier to effective parenteral migraine-specific therapy. Paradoxically, the characteristics believed to be responsible for the high rate of efficacy seen with the injectable forms — faster bioavailability and greater systemic exposure compared with oral formulations — may also contribute to the relatively high rate of triptan sensations. It has been suggested that novel sumatriptan formulations with pharmacokinetics similar to SC sumatriptan are promising for use in clinical practice [2].

In this study of subjects with rapidly-escalating attacks of episodic migraine, treatment with DFN-11, a 3 mg SC sumatriptan autoinjector, was comparable to SC sumatriptan 6 mg on all clinically relevant efficacy endpoints. The pain-free results at 60 min postdose suggest that DFN-11 may be a fast-acting alternative to commercial formulations of injectable sumatriptan, especially for patients who awaken with full-blown attacks already in progress [19]. In our study, the lower, 3-mg dose of sumatriptan in DFN-11 did not appear to impede the overall therapeutic effect, and DFN-11 maintained an efficacy timecourse similar to SC sumatriptan 6 mg — a finding in accord with a previous report comparing 3 and 6 mg SC sumatriptan [20].

The most common AEs were triptan sensations known to be associated with SC triptan usage; all were mild to moderate and similar in frequency and severity to those seen in previous studies of the SC formulation of sumatriptan. Notably, while most subjects experienced symptoms with both sumatriptan doses, fewer subjects had triptan sensations only after treatment with the 3-mg dose than with the 6-mg dose. In addition, the triptan sensation of chest pain, which has been shown to cause up to 10% of sumatriptan-treated patients to discontinue therapy [21] and may lead to substantially increased medical costs for migraine care [22], was not observed after the 3-mg dose of DFN-11 treatment. With the 6 mg SC sumatriptan treatment, 10% of subjects were affected, and the mean duration of the events exceeded 6 h. The implications of these findings deserve to be explored in future research.

While the results with DFN-11 are similar in many aspects to those seen in previous dose-comparing studies of SC sumatriptan, there may be clinically important differences. For example, an open-label trial comparing the 3 and 6 mg SC doses (in which 80% of subjects preferred the 3-mg dose) found, as in the current study, that the proportions of subjects with pain-free responses were similar between the 2 doses at 1 and 2 h after treatment [20]. However, the 1-h pain relief rate for the 3-mg SC dose in this study exceeded previous reports for the 6-mg SC dose at that timepoint (83% vs 70%) [14]. The higher treatment response may reflect the benefits of treating at the first sign of migraine pain, as subjects in the earlier studies [14, 20] waited for pain to reach moderate intensity. AEs were overall slightly more frequent with the 6-mg dose than with the 3-mg dose [14, 20].

The current study has strengths and limitations. Its main strength is its originality; this is the first well-controlled study to compare the efficacy and tolerability of 3- and 6-mg SC doses of sumatriptan. Another strength is that because subjects in this study treated at the first sign of migraine, which can prevent the development of central sensitization [23] and optimize pain-free efficacy [24–26], the performance of DFN-11 was assessed under conditions that simulate actual treatment scenarios. Limitations of the study include the lack of power and sample size calculation, as well as the small sample size, which restrict the validity and generalizability of results. It is also possible that outcomes might have been affected by subjects’ using 2 consecutive injections to provide a 6-mg dose of sumatriptan, but there is no evidence that this method of administering study medication influences response to sumatriptan.

## Conclusions

In this randomized, double-blind, crossover pilot study comparing 3 mg SC sumatriptan with 6 mg SC sumatriptan for acute treatment of rapidly-escalating attacks of episodic migraine, both the 3- and 6-mg doses demonstrated comparable efficacy on all efficacy endpoints. The 3-mg and 6-mg SC doses were also similar on safety parameters, but DFN-11 was associated with better tolerability, as shown by a lower incidence of triptan sensation AEs. Of particular interest was the lack of chest pain as an AE with DFN-11. These preliminary results need to be confirmed in larger clinical studies.

## Abbreviations

AE: Adverse event
ALT: Alanine aminotransferase
ANOVA: Analysis of variance
AST: Aspartate aminotransferase
ECG: Electrocardiogram
ICHD: International Classification of Headache Disorders
MAO-A: Monoamine oxidase A
SC: Subcutaneous
SD: Standard deviation
ULN: Upper limit of normal
